# Phosphatidylethanolamine Induces an Antifibrotic Phenotype in Normal Human Lung Fibroblasts and Ameliorates Bleomycin-Induced Lung Fibrosis in Mice

**DOI:** 10.3390/ijms19092758

**Published:** 2018-09-14

**Authors:** Luis G. Vazquez-de-Lara, Beatriz Tlatelpa-Romero, Yair Romero, Nora Fernández-Tamayo, Fernando Vazquez-de-Lara, Jaime M. Justo-Janeiro, Mario Garcia-Carrasco, René de-la-Rosa Paredes, José G. Cisneros-Lira, Criselda Mendoza-Milla, Francesco Moccia, Roberto Berra-Romani

**Affiliations:** 1 Facultad de Medicina, Benemérita Universidad Autónoma de Puebla, Puebla 72410, Mexico; beatlarom@outlook.com (B.T.-R.); yair12@hotmail.com (Y.R.); norafertamayo@gmail.com (N.F.-T.); fvazdela@gmail.com (F.V.-d.-L.); mgc30591@yahoo.com (M.G.-C.); rberra001@hotmail.com (R.B.-R.); 2 Hospital General del Sur, Puebla 72490, Mexico; jaime_justo@hotmail.com (J.M.J.-J.); rdelarosa2000@hotmail.com (R.d.-l.-R.P.); 3 Instituto Nacional de Enfermedades Respiratorias “Ismael Cosío Villegas”, México City 14080, Mexico; jgclira@yahoo.com.mx (J.G.C.-L.); criselda.mendoza@gmail.com (C.M.-M.); 4 Laboratory of General Physiology, Department of Biology and Biotechnology ‘‘Lazzaro Spallanzani”, University of Pavia, 27100 Pavia, Italy; francesco.moccia@unipv.it

**Keywords:** pulmonary surfactant, phosphatidylethanolamine, lung fibrosis, bleomycin model, lung fibroblasts, Ca^2+^ signaling

## Abstract

Lung surfactant is a complex mixture of phospholipids and specific proteins but its role in the pathogenesis of interstitial lung diseases is not established. Herein, we analyzed the effects of three representative phospholipid components, that is, dipalmitoilphosphatidylcoline (DPPC), phosphatidylglycerol (PG) and phosphatidylethanolamine (PE), on collagen expression, apoptosis and Ca^2+^ signaling in normal human lung fibroblasts (NHLF) and probed their effect in an experimental model of lung fibrosis. Collagen expression was measured with RT-PCR, apoptosis was measured by using either the APOPercentage assay kit (Biocolor Ltd., Northern Ireland, UK) or the Caspase-Glo 3/7 assay (Promega, Madison, WI, USA) and Ca^2+^ signaling by conventional epifluorescence imaging. The effect in vivo was tested in bleomycin-induced lung fibrosis in mice. DPPC and PG did not affect collagen expression, which was downregulated by PE. Furthermore, PE promoted apoptosis and induced a dose-dependent Ca^2+^ signal. PE-induced Ca^2+^ signal and apoptosis were both blocked by phospholipase C, endoplasmic reticulum pump and store-operated Ca^2+^ entry inhibition. PE-induced decrease in collagen expression was attenuated by blocking phospholipase C. Finally, surfactant enriched with PE and PE itself attenuated bleomycin-induced lung fibrosis and decreased the soluble collagen concentration in mice lungs. This study demonstrates that PE strongly contributes to the surfactant-induced inhibition of collagen expression in NHLF through a Ca^2+^ signal and that early administration of Beractant enriched with PE diminishes lung fibrosis in vivo.

## 1. Introduction

A common pathological feature in acute lung injury and diverse interstitial lung diseases, such as idiopathic pulmonary fibrosis (IPF), is the migration of fibroblasts and myofibroblasts from the interstitium to the alveolar spaces [1,2,3]. When the lung alveolar epithelium becomes injured and the basement membrane loses its integrity, an extravasation of plasma-derived fluid takes place and activated alveolar epithelial cells chemoattract mesenchymal cells to the alveolar regions [4]. In the process of migration through partially disrupted and denuded epithelial basement membranes, fibroblasts are exposed to the components of alveolar spaces including surfactant lipids and proteins.

The lipids of lung surfactant mainly include phospholipids (PL) (~90–95 wt %) and a small amount of neutral lipids (~5–10 wt %), such as cholesterol. Phosphatidylcholine (PC) accounts for ~80% of the total PL content, whereas the remaining 20% consists primarily of unsaturated anionic PL (e.g., lyso-bis-phosphatidic acid, phosphatidylinositol and phosphatidylglycerol (PG)), which account for ~15% of the total PL and by small amounts of non-PC zwitterionic PL (e.g., sphingomyelin and phosphatidylethanolamine (PE)) [5]. Beractant (Survanta™, Abbvie Inc., North Chicago, IL, USA) is a modified bovine pulmonary surfactant organic extract, to which synthetic dipalmitoilphosphatidylcoline (DPPC), triacylglycerol and palmitic acid are added. Beractant is widely used for the treatment of respiratory distress syndrome in premature newborns [6]. In a previous study, we showed that pulmonary surfactant promotes programmed cell death of normal human lung fibroblasts and induces the upregulation of matrix metalloproteinase-1 and the downregulation of type I collagen [7]. In that study, we used Beractant, a natural bovine lung extract containing phospholipids, neutral lipids, fatty acids and surfactant-associated hydrophobic proteins B and C. In other study [8], we found that Beractant promoted apoptosis and reduced the expression levels of type I collagen through an increase in intracellular Ca^2+^ concentration ([Ca^2+^]_i_) that was initiated by the recruitment of phospholipase C (PLC). Intriguingly, neither DPPC nor PG were able to induce an increase in [Ca^2+^]_i_, which suggests that other surfactant components induce apoptosis and regulate gene expression in normal lung fibroblasts (NHLF).

The aim of this work was to test the effect of three representative phospholipids on the expression of collagen in NHLF: DPPC (PC class), PG (anionic PL) and PE (non-PC zwitterionic PL) and to test this effect in vivo in an experimental model of lung fibrosis. Evidence is presented that physiological concentrations of PE downregulate collagen expression and induce apoptosis in NHLF. We also provide evidence that PE elicits an increase in [Ca^2+^]_i_ and that the pharmacological blockade of this Ca^2+^ signal attenuates type I collagen expression and apoptosis. Finally, we also show that Beractant enriched with PE ameliorates lung fibrosis in bleomycin-induced lung injury in mice.

## 2. Results

### 2.1. Effect of Different Phospholipid Components of Surfactant on Collagen mRNA Expression

We have previously demonstrated that surfactant reduced the expression of collagen by human lung fibroblasts [7]. To elucidate which components of surfactant participate in this process, we evaluated the effect of three representative phospholipids that are found in normal pulmonary surfactant: DPPG (PC class), PG (anionic PL) and PE (non-PC zwitterionic PL).

**Figure 1:**
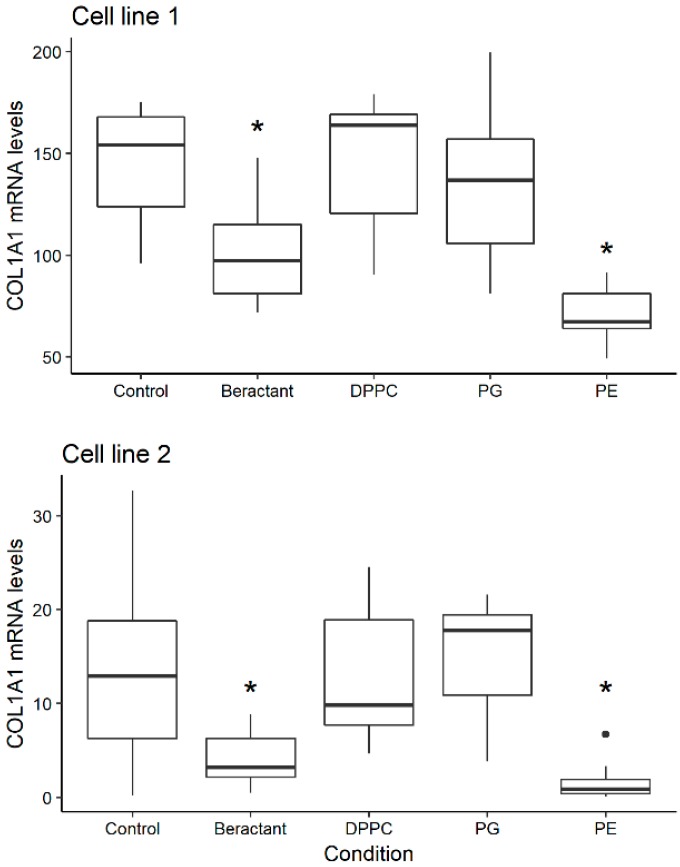
Effect of dipalmitoilphosphatidylcoline (DPPC), phosphatidylglycerol (PG) and phosphatidylethanolamine (PE) on collagen expression in lung fibroblasts. Cells from passages 5–10 were placed in 6 well plates and when confluence was reached, culture medium was substituted with serum-free F-12 for 24 h. Beractant (500 µg·mL^−1^) and corresponding phospholipids were diluted in F-12 without serum and cells were incubated for 48 h. Controls were grown for the same time in F-12 without serum. DPPC: 200 µg·mL^−1^; PG: 50 µg·mL^−1^; PE: 50 µg·mL^−1^. The figure represents the pooled data of two independent experiments in two cell lines. Asterisks denote significant differences (*p* < 0.05, ANOVA with post-hoc Dunnett test against the control).

In the previous work, we saw that Beractant at 500 mg mL^−1^ decreases collagen expression in normal human lung fibroblasts. Considering the proportions of lipids reported in lung surfactant [5], DPPC was used at a dose of 200 µg·mL^−1^ and PG and PE were added at a concentration of 50 µg·mL^−1^. Lung fibroblasts from passages 5–10 were placed in 6 well plates and when confluence was reached, culture medium was substituted with serum-free F-12 for 24 h. The phospholipids were added in triplicates and cells were incubated for 48 h. Culture medium was aspirated and RNA was isolated. As illustrated in Figure 1, no effect was observed with DPPC and PG. In contrast, collagen expression decreased significantly (cell line 1: 146.8 ± 28.3 vs. 68.3 ± 18.7; cell line 2: 12.9 ± 9.6 vs. 1.6 ± 1.9) with PE at 50 µg·mL^−1^ (*p* < 0.05, ANOVA with post-hoc Dunnett Test). As shown elsewhere [7], Beractant (500 µg·mL^−1^) also reduced collagen mRNA expression in NHLF.

### 2.2. Treatment of NHLF with PE Causes a Dose- and Time-Dependent Decrease of Collagen Expression

**Figure 2:**
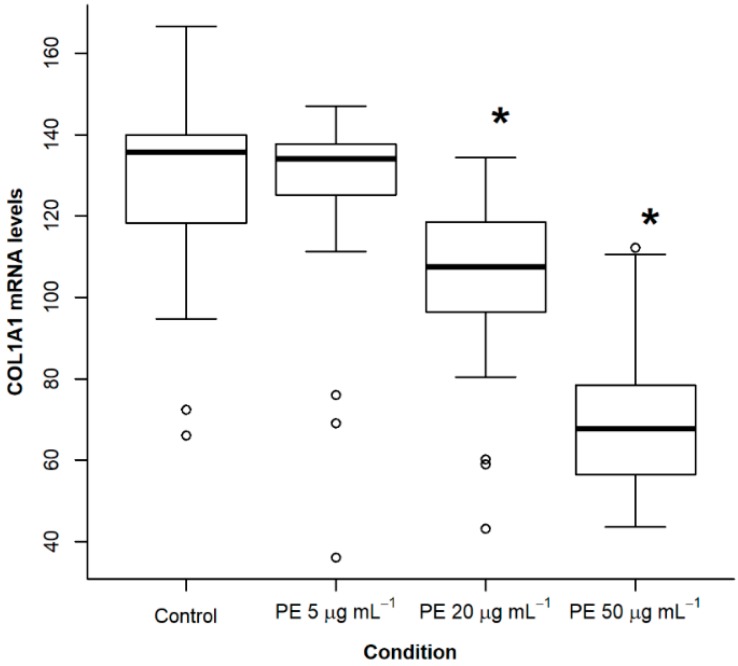
Effect of different concentrations of phosphatidylethanolamine (PE) on collagen expression in normal human lung fibroblasts. Cells from passages 5–10 were placed in 6 well plates and when confluence was reached, culture medium was substituted with serum-free medium for 24 h. PE was diluted in F-12 without serum at 5, 20 and 50 µg·mL^−1^ and cells were incubated for 48 h. Controls were grown for the same time in F-12 without serum. The figure represents the pooled data of three independent experiments. Asterisks denote significant differences (*p* < 0.05, ANOVA with post-hoc Dunnett test against the control).

**Figure 3:**
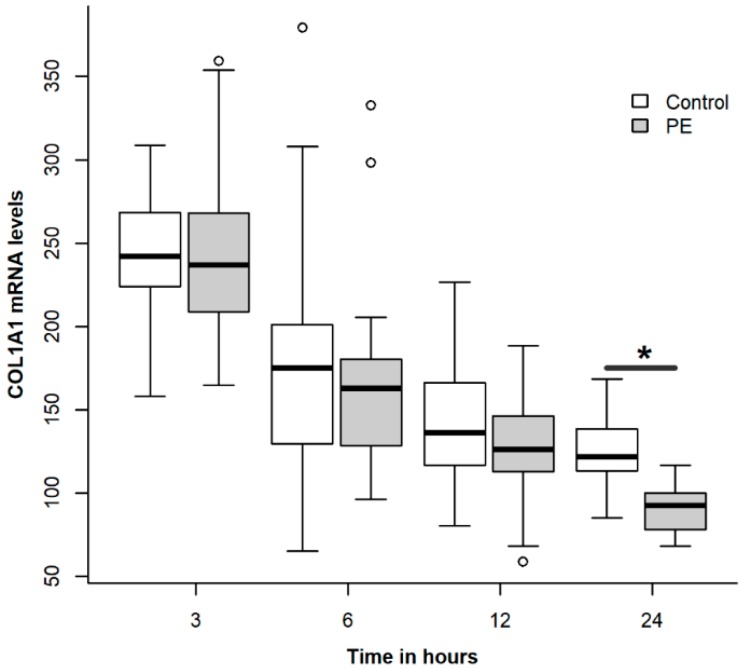
Effect of incubation time of phosphatidylethanolamine (PE) on collagen expression in normal human lung fibroblasts. Cells from passages 5–10 were placed in 6 well plates and when confluence was reached, culture medium was substituted with serum-free medium for 24 h. PE was diluted in F-12 without serum at 50 µg·mL^−1^ and cells were incubated for 3, 6, 12 and 48 h. Controls were grown for the same time in F-12 without serum. Asterisk indicates a significant difference when compared with the corresponding control (*p* < 0.05, Student *t*-test).

Since PE produced a significant and consistent reduction in the expression of collagen in relation to the other phospholipids tested, we performed a dose response experiment within physiologic ranges. Fibroblasts were incubated with PE at doses of 5, 20 and 50 µg·mL^−1^ for 48 h. At a dose of 5 µg·mL^−1^, PE did not have a significant effect. However, at doses of 20 and 50 µg·mL^−1^, a significant decrease was observed in collagen expression (*p* < 0.05; Figure 2). We also tested different incubation times with PE at 50 µg·mL^−1^ and observed that a significant decrease in collagen expression occurred at 24 h (*p* < 0.05, ANOVA with post-hoc Dunnett test, Figure 3).

### 2.3. Effect of PE on Fibroblast Apoptosis

**Figure 4:**
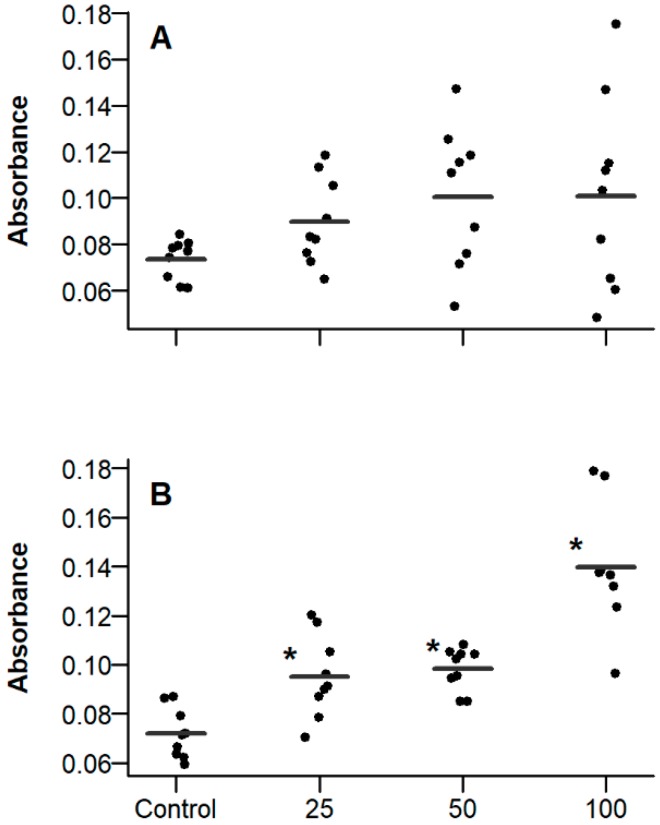
Effect of phosphatidylethanolamine (PE) on apoptosis of normal human lung fibroblasts. Forty-eight well plates were seeded with 40,000 cells per well and incubated for 24 (**A**) or 48 h (**B**) with 25, 50 or 100 µg·mL^−1^ PE in free-serum medium. Apoptosis was measured with the APOPercentage apoptosis assay (Biocolor). Lines represent the mean of the groups. Asterisks indicate significant differences (*p* < 0.05, ANOVA with post-hoc Dunnett test against the control).

We have previously observed that Beractant induces fibroblast apoptosis [7], which represents an additional strategy to hamper collagen deposition and ameliorate pulmonary fibrosis [9]. Accordingly, we tested if this effect could be reproduced by PE. Figure 4 shows the effect of different concentrations of PE at 24 and 48 h. Apoptosis was not different from control when cells were incubated for 24 h with 25, 50 or 100 µg·mL^−1^ (Figure 4A); when fibroblasts were incubated for 48 h (Figure 4B), a significant difference in apoptosis was observed starting from a PE concentration of 25 µg·mL^−1^ (*p* < 0.05, ANOVA with post-hoc Dunnett test). The figure represents the pooled data of three independent experiments.

### 2.4. A Calcium Signal Is Elicited by PE and a Phospholipase C Inhibitor Decreases PE Effect on Collagen Expression and Fibroblast Apoptosis

**Figure 5:**
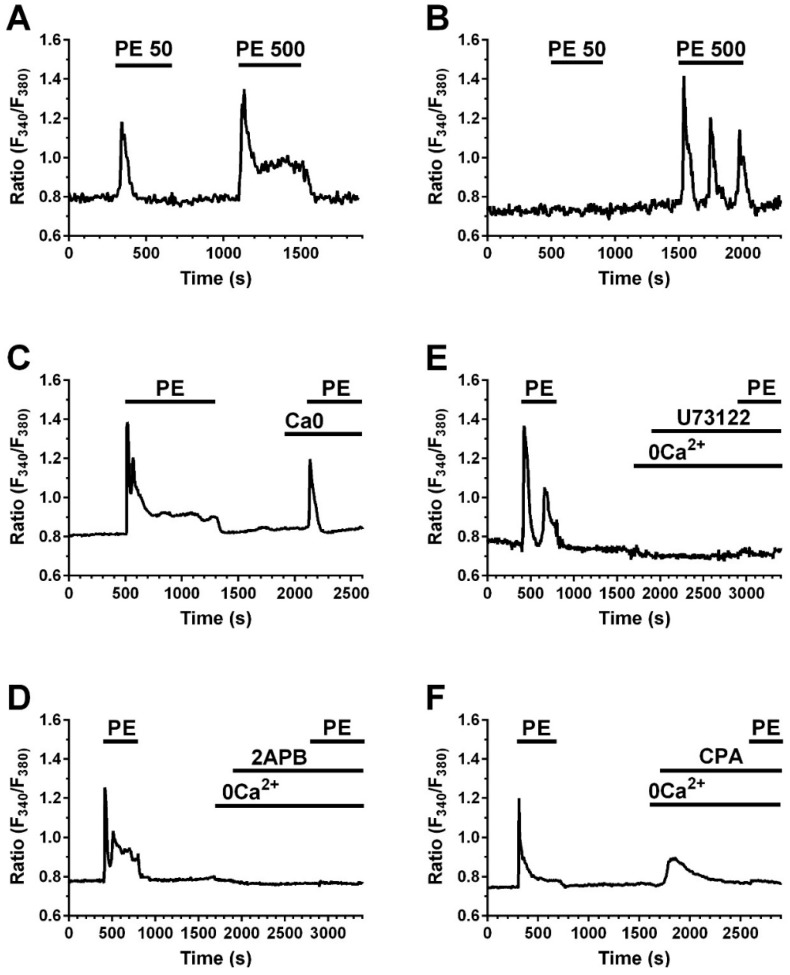
Phosphatidylethanolamine (PE) induced Ca^2+^ signals in normal human lung fibroblasts. (**A**) Representative tracing of a cell that displayed a Ca^2+^ signal in response to both 50 and 500 µg·mL^−1^ of PE; (**B**) Representative tracing of a cell that did not respond to PE 50 µg·mL^−1^; (**C**) Representative Ca^2+^ tracing induced by PE 500 µg·mL^−1^ in the presence and absence of extracellular Ca^2+^ (0Ca^2+^); (**D**) U73122 10 µM prevents the Ca^2+^ response to PE 500 µg·mL^−1^; (**E**) 2APB 50 µM prevents the Ca^2+^ response to PE 500 µg·mL^−1^; (**F**) CPA 10 µM, abolished the Ca^2+^ response to PE 500 µg·mL^−1^ under 0Ca^2+^ conditions.

**Figure 6:**
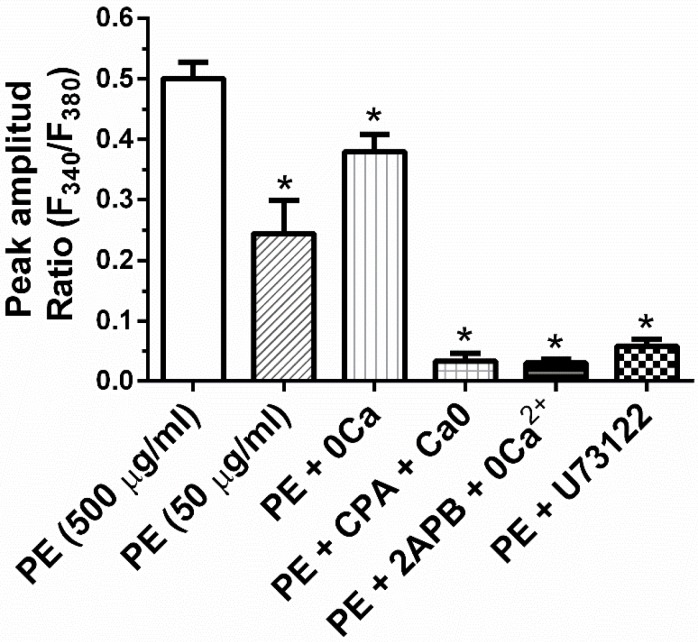
Statistical analysis of Ca^2+^ imaging experiments. Mean ± SE of the amplitude of the Ca^2+^ response to PE 500 µg·mL^−1^ under the designated treatments. Asterisks denote significant differences (*p* < 0.05, ANOVA with *post-hoc* Dunnett test against the control).

**Figure 7:**
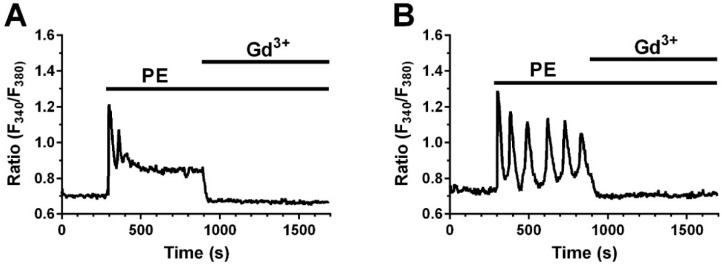
Gd^3+^ blocks phosphatidylethanolamine (PE)-induced Ca^2+^ plateau and Ca^2+^ oscillations in NHLF. (**A**) Addition of Gd^3+^ (5 µM) inhibited the sustained plateau phase of the Ca^2+^ signal induced by PE (500 µg·mL^−1^); (**B**) Application of La^3+^ (5 μM) inhibited PE-elicited Ca^2+^ oscillations (500 µg·mL^−1^).

We have previously shown that Beractant elicits an intracellular Ca^2+^ signal in NHLF and that this Ca^2+^ response was not triggered either by DPPC or PG [8]. Thus, we tested if PE mimics this effect. NHLF were loaded with the Ca^2+^-sensitive fluorochrome, Fura-2 and then stimulated with PE at increasing doses and in the absence and in the presence of specific inhibitors of the Ca^2+^ signaling toolkit. PE induced an increase in [Ca^2+^]_i_ similar to that evoked by Beractant (Figure 5) [8]. At 50 µg· mL^−1^, around 20% of cells responded with a transient Ca^2+^ spike (Figure 5A, left); 500 µg·mL^−1^ PE induced a biphasic Ca^2+^ signal in all the cells, which consisted in an initial Ca^2+^ spike followed by a plateau phase of intermediate amplitude (Figure 5A, right) or Ca^2+^ oscillations (Figure 5B, left). The Ca^2+^ response to Beractant was mediated by the interaction between inositol-1,4,5-trisphosphate (InsP_3_)-dependent Ca^2+^ release from the endoplasmic reticulum (ER) and store-operated Ca^2+^ entry (SOCE) [8]. Likewise, upon removal of extracellular Ca^2+^ (0Ca^2+^) PE induced a transient increase in [Ca^2+^]_i_ which lacked the plateau or oscillation phase (Figure 5C). This finding confirms that, similar to Beractant, the Ca^2+^ response to PE is initiated by endogenous Ca^2+^ release and sustained by SOCE during the following plateau or oscillation phase. To assess this hypothesis, we pre-incubated the cells with the aminosteroid U73122 (10 µM, 30 min), a rather selective PLC blocker [5], or with 2-aminoethoxydiphenyl borate (2APB) (50 µM, 30 min), which selectively targets InsP_3_ receptors (InsP_3_Rs) under 0Ca^2+^ conditions [10]. As shown in Figure 5D and in Figure 5E, respectively, either U73122 or 2APB blocked Ca^2+^ response to PE. Furthermore, PE-induced increase in [Ca^2+^]_i_ was prevented by cyclopiazonic acid (CPA; 10 µM), a specific inhibitor of Sarco-Endoplasmic Reticulum Ca^2+^-ATPase (SERCA) activity, which depletes the ER Ca^2+^ content in NHLF [8], Figure 5F. The statistical analysis is reported in Figure 6. To evaluate the participation of SOCE on the Ca^2+^ signaling evoked by PE, we applied 5 µM of Gd^3+^, a concentration which selectively hinders store-operated channels [8,11]. Similar to what was reported for Beractant [8], Gd^3+^ interrupt both the prolonged plateau phase (Figure 7A) and the repetitive Ca^2+^ oscillations (Figure 7B) evoked by PE. Collectively, these finding strongly suggest that PE is the phospholipid component that actually triggers the Ca^2+^ response so surfactant in NHLF and that this Ca^2+^ signal arises downstream of PLC activation.

**Figure 8:**
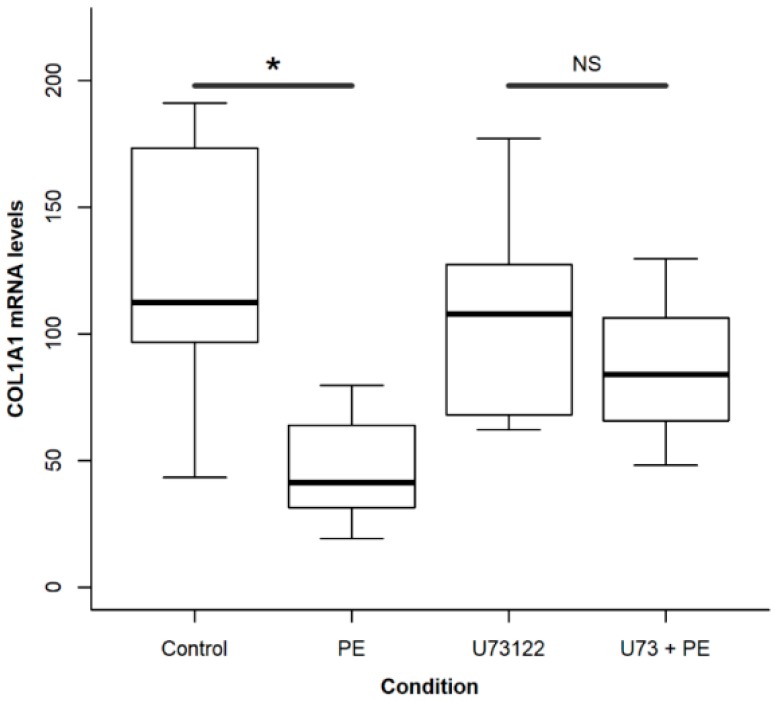
Influence of U73122 on phosphatidylethanolamine (PE)-induced collagen expression. Cells grown in six well plates were incubated for 48 h in serum-free culture medium with PE 50 µg mL^−1^, either in the presence or absence of U73122 10 µM in dimethyl sulfoxide (DMSO). Controls were incubated with the same amount of DMSO used in the experimental wells. Individual comparisons were made for each pair of control and experimental groups with Student *t* test. Asterisk indicates a *p* < 0.05, NS: non-significant.

To test if there is a relationship between the Ca^2+^ signal and the PE effect on collagen expression, cells were incubated in the presence of PE with or without U73122 (10 µM, 30 min) for 48 h to prevent both InsP_3_-dependent Ca^2+^ release and SOCE activation. The decrease in collagen expression provoked by PE was significantly attenuated in the presence of U73122 (*p* < 0.05, Student *t* test, Figure 8), thereby confirming our previous data with Beractant [8].

**Figure 9:**
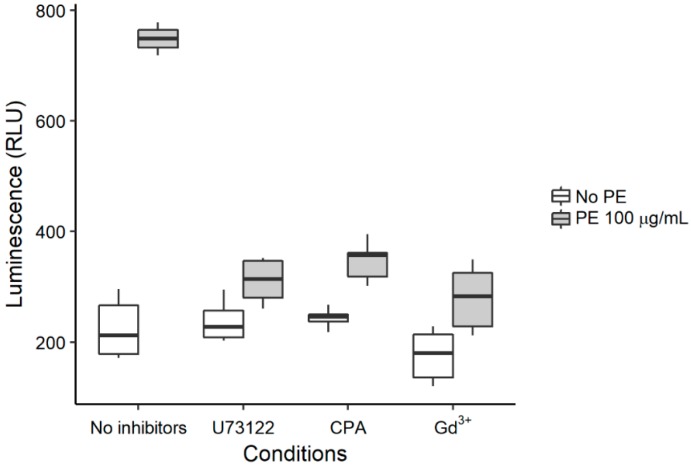
Inhibition of calcium signaling prevents phosphatidylethanolamine (PE)-induced apoptosis. Ninety-six well plates were seeded with 20,000 cells and incubated for 48 h in free serum medium with 100 µg·mL^−1^ PE in the absence or in the presence of U73122 (10 µM), CPA (5 µM) and gadolinium (5 µM). Apoptosis was measured by using the Caspase-Glo 3/7 assay (Promega). Asterisks indicate significant differences (*p* < 0.05, ANOVA with *post-hoc* Dunnett test against the control).

Finally, we assessed whether there is a relationship between the Ca^2+^ signal and the PE effect on apoptosis. We found that PE induced apoptosis when administered at 100 µg·mL^−1^ to human lung fibroblasts under control conditions for 48 h (Figure 9). However, the apoptotic process was significantly (*p* < 0.05) inhibited when the accompanying Ca^2+^ signal was impaired by using any of the following drugs: U73122 10 µM, CPA 5 µM and Gd^3+^ 5 µM. These data, therefore, endorse the view that PE uses intracellular Ca^2+^ signaling to promote apoptosis in human lung fibroblasts.

### 2.5. Phosphatidylethanolamine Mitigates Bleomycin-Induced Lung Fibrosis

Since PE strongly reduces the expression of collagen in fibroblast in vitro, we examined whether this phospholipid may have an effect on the development of lung fibrosis in vivo. For this purpose, mice were challenged with bleomycin and the lung fibrotic response was analyzed at 21 days. Five groups of animals were studied. Number of animals and treatment protocols are summarized in Table 1. All treatments were administered every 48 h for 6 doses, starting 2 h after bleomycin instillation via aerosol as described in Methods. Beractant was enriched with PE sonicated in normal saline at a final concentration of 0.75 mg·mL^−1^. The rationale was that PE comprises approximately 3% of surfactant phospholipids [5] and the intention was to duplicate the amount of PE. Twenty-one days after bleomycin instillation lungs were obtained, the right lung was used to measure soluble collagen content and the left lung for histological analysis.

**Figure 10:**
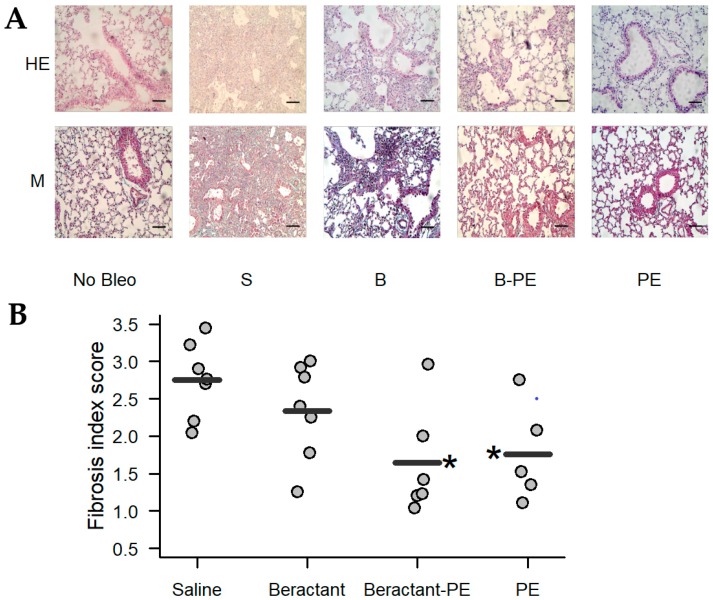
Effect of 1.5 mL of inhaled Beractant, phosphatidylethanolamine (PE)-enriched Beractant and PE on pulmonary fibrosis in bleomycin treated C57/BL mice. The first dose was given the same day of bleomycin administration. **Saline**: after intratracheal administration of 3 U/Kg bleomycin, 1.5 mL of normal saline was nebulized every 48 h for 6 doses. **Beractant**: after the administration of bleomycin, Beractant 400 mg/Kg were nebulized every 48 h for 6 doses. **Beractant-PE**: the treatment consisted of 400 mg·mL^−1^ of Beractant enriched with PE at a final concentration of 0.75 mg mL^−1^. **PE**: treatment consisted of PE 0.75 mg·mL^−1^. (**A**) Representative photomicrographs from experimental groups (scale bar, 150 µm). Lung tissue samples were stained with hematoxylin-eosin (HE) and Masson (M). No bleo: control with no bleomycin and saline treatment; S: control with inhaled saline; B: inhaled Beractant; B-PE: inhaled PE-enriched Beractant; PE: inhaled PE; (**B**) Semi quantitative evaluation of lung lesions. Asterisks indicate a significant difference (*p* < 0.05, ANOVA with *post-hoc* Dunnett test against the control (Saline).

**Figure 11:**
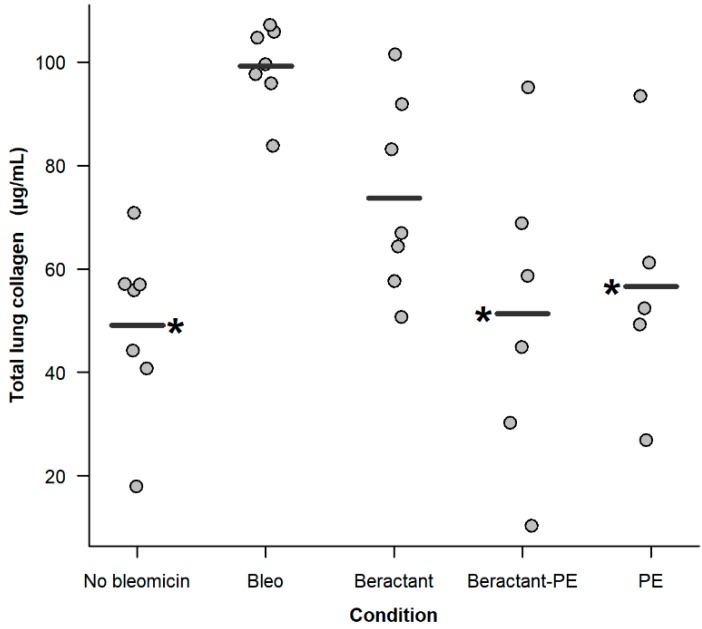
Effect of 1.5 mL of inhaled Beractant, phosphatidylethanolamine (PE)-enriched Beractant and PE on total lung collagen content in bleomycin treated C57/BL mice. The first dose was given the same day of bleomycin administration. **Bleo**: after intratracheal administration of 4 U/Kg bleomycin, 1.5 mL of normal saline was nebulized every 48 h for 6 doses. **Beractant**: after the administration of bleomycin, Beractant 400 mg/Kg were nebulized every 48 h for 6 doses. **Beractant-PE**: the treatment consisted of 400 mg mL^−1^ of Beractant enriched with PE at a final concentration of 0.75 mg mL^−1^. **PE**: treatment consisted of phosphatidlethanolamine 0.75 mg mL^−1^. Lines represent the mean of the groups. Total collagen content was measured with the Sircoll assay (Biocolor Ltd., Northern Ireland, UK). (* *p* < 0.05, ANOVA with *post-hoc* Dunnett test against the saline-treated group).

As illustrated in Figure 10A, morphological analysis of the lungs showed that surfactant enriched with PE and PE decreased lung inflammation and fibrosis induced by bleomycin. The effect on the fibrotic response was corroborated by the semi quantitative analysis of the fibrotic index (Figure 10B). This finding was consistent with the quantification of collagen. As shown in Figure 11, bleomycin mice almost doubled the concentration of collagen in the lungs when compared to the control group challenged with normal saline solution (99.2 ± 8.1 versus 49.1 ± 16.9 µg/mL; *p* < 0.05). Treatment with Beractant tended to decrease the concentration of collagen but the difference was not statistically significant (73.7 ± 18.8; *p* < 0.05). Treatment of the bleomycin-injured mice with PE (56.6 ± 24.2 µg/mL; *p* < 0.05) or, even more, with surfactant enriched with PE (51.2 ± 29.7 µg/mL; *p* < 0.05) showed a significant decrease in lung collagen accumulation. All comparisons were made with ANOVA and *post-hoc* Dunnett test against the bleomycin-injured mice treated with normal saline.

## 3. Discussion

Lung surfactant is a complex mixture of phospholipids and specific proteins that has many essential functions. Its role in lung fibrosis has not been clearly established but there is some evidence in experimental models that modifications in the concentration and/or composition of surfactant are present when lung fibrosis occurs [12].

In the clinical context, some interstitial lung diseases, such as sarcoidosis, idiopathic pulmonary fibrosis and hypersensitivity pneumonitis, are associated with decreased phospholipid content of surfactant [13]. Likewise, increased levels of SP-A have been described in untreated patients with idiopathic pulmonary fibrosis (IPF) and hypersensitivity pneumonitis [14]. Even though the mechanism has not been clearly established, it has been shown that SP-A interacts differentially with surfactant phospholipids, both in the polar group and the fatty acid chains in order to form aggregates [15] suggesting that an increase in SP-A could favor alterations in the relative composition of the lipid fraction.

We have previously shown that Beractant decreases collagen expression in normal human lung fibroblasts through an increase in [Ca^2+^]_i_ [7,8]. Type I collagen represents the major fibrous collagen synthesized by wound fibroblasts during the repair process and is exceedingly deposited in response to a traumatic injury, thereby leading to lung fibrosis [16,17]. In the present study, we found that when DPCC, PG and PE were used individually at physiological concentrations, only PE was able to diminish collagen expression and this effect was time and dose-dependent. The exact mechanism of action remains to be elucidated. In neutrophils, there is evidence that pulmonary surfactant could act through the incorporation of ionic channels in the membrane, producing depolarization and activation of G coupled receptor which produce intracellular Ca^2+^ release [18]. Furthermore, we previously showed that Beractant caused an increase in [Ca^2+^]_i_ that was patterned by the interplay between InsP_3_-dependent ER [Ca^2+^]_i_ release and SOCE [8]. In that study, we found that neither DPCC nor PG were able to elevate [Ca^2+^]_i_. Herein, we demonstrate that PE elicits an intracellular Ca^2+^ signal in lung fibroblasts that mimics the Ca^2+^ response to Beractant. Accordingly, PE induced different patterns of intracellular Ca^2+^ signals, including a transient Ca^2+^ spike, a biphasic Ca^2+^ elevation and intracellular Ca^2+^ oscillations. Moreover, the onset of PE-induced intracellular Ca^2+^ signals was prevented by inhibiting the PLC/InsP_3_ signaling pathway with U73122 or 2-APB and was sustained by SOCE. Therefore, these findings lend credit to the notion that PE is the major phospholipid component that underlies the Ca^2+^ response to Beractant. Furthermore, the effect of PE on collagen expression was attenuated by U73122. This is in accordance with our findings reported previously with Beractant and confirms that PE regulates gene expression in NHLF through an increase in [Ca^2+^]_i_ [8]. Accordingly, intracellular Ca^2+^ signaling represents an established mechanism to control gene expression in mammalian cells [19,20,21]. Moreover, an increase in [Ca^2+^]_i_ has been shown to regulate collagen expression also in cultured human cardiac [22,23] and pulmonary [24] fibroblasts as well as in human glomerular mesangial cells [25]. Finally, we found that PE-induced apoptosis was prevented by interfering with the accompanying Ca^2+^ signal by pre-treating the cells with U73122 to block PLC, CPA to deplete the ER Ca^2+^ store and Gd^3+^ to inhibit SOCE. These data confirm the finding that beractant induces apoptosis in a Ca^2+^-dependent manner in human ling fibroblasts [8].

However, we cannot rule out alternative mechanisms to explain the effect of PE on collagen expression in NHLF. For instance, PE is the main phospholipid that favors the negative curvatures of the cell membrane. It is also a molecule with a net neutral charge due to its amino group (zwitterionic). Previous works have studied the interaction of phospholipids with integral membrane proteins. The first factor that could be modified by phospholipids is the hydrophobic width of the lipid bilayer which is determined by the fatty acid chains of phospholipids. This modification determines how much of the protein is included in the membrane and what part is responsible for interaction. In addition, the polar group can interact with the protein and change its conformation [26,27]. Future work is mandatory to assess whether this mechanism is somehow involved in PLC recruitment by PE. Moreover, PE has been shown to activate the extracellular signal-regulated kinases (Erk) and Stat signaling pathways [28], which may inhibit type 1 collagen expression in human dermal fibroblasts [29,30]. Of note, intracellular Ca^2+^ signaling may recruit both Erk [31] and Stat signaling [32].

The deposition of excess collagen in tissues is the mainstay in the pathogenesis of fibrosis. Drugs that inhibit fibroblast proliferation and collagen expression in vitro potentially have a therapeutic effect. In preclinical studies with pirfenidone and nintedanib, currently in clinical use, a decrease in lung fibroblast proliferation and reduced collagen expression was shown [33]. In view of this, we investigated the effect of aerosolized Beractant, PE-enriched Beractant and PE alone in C57/BL mice with bleomycin-induced lung fibrosis. Our results show that Beractant enriched with PE reduces the fibrotic response induced by bleomycin in vivo. More importantly, PE alone displayed a similar effect to that of PE-enriched Beractant, thereby supporting the notion that this molecule plays a pivotal role in the antifibrotic properties of surfactant. Future work will have to confirm that intracellular Ca^2+^ signaling plays a crucial role in reducing fibroblast proliferation and collagen expression also in vivo. In addition to the molecular mechanisms previously proposed, PE could also act in vivo by modifying the biophysical properties of surfactant. Rapid adsorption of lipids to the air-liquid interface is an essential property of lung surfactant and the hydrophobic surfactant proteins SP-B and SP-C play a crucial role in this effect but recently it has been reported that PE also induces acceleration in the adsorption rate [34,35]. In other experiments, aerosolized nanovesicles prepared with DPPC and dioleylphosphatidylethanolamine improved the resistance of pulmonary surfactants to inhibition in mice with acid-induced lung injury [36]. In our model, these effects could be acting in concert to avoid the alveolar rupture and epithelial damage caused by bleomycin.

However, it is worth noting that the antifibrotic effects are limited to the early administration of exogenous surfactant in the bleomycin model. We did not see such effect when administered at 48 h after the insult (data not shown). Proving different doses of enrichment and using a different animal model would be required in order to establish whether PE enriched surfactant may be a possible treatment for fibrosing diseases of the lung. This work, in turn, calls for future investigations that would elucidate the molecular mechanisms responsible for the effect of PE.

## 4. Materials and Methods

### 4.1. Isolation and Purification of Normal Human Lung Fibroblasts

NHLF were obtained from kidney donors with brain death upon previous consent of the family, clearly stating that the lung tissue will be used to extract cells for research purposes and data will remain anonymous. The protocol was accepted by the ethical boards of the Hospital General de Puebla and the Benemérita Universidad Autónoma de Puebla College of Medicine. After clamping the aorta, a lung sample was obtained from the left lower lobe and placed in HAM F-12 medium. The tissue was processed with Trypsin-EDTA solution 1× (Sigma, St. Louis, MO, USA) and serum-free F-12 medium. The filtrate was centrifuged at 200 g for 10 min and the pelleted cells were re-suspended in F-12 (10% fetal bovine serum) and layered in T-25 flasks. Cells were grown to a 75% confluence in F-12 medium, at 37 °C on an atmosphere of 95% of air and 5% of CO_2_. For the experiments, cells from passages 5 to 10 were placed in 6 well plates and when confluence was reached, culture medium was substituted with serum-free F-12 for 24 h. DPPC, PG, PE (all from Sigma-Aldrich) or Beractant (Abbot) were used at different concentrations. Three cell lines were used for all the experiments, obtained from a thirty-year-old male, a sixty-one-year-old male and a fifteen-year-old female.

### 4.2. RNA Isolation and RT-PCR Analysis

Total RNA extraction was performed by the TRIzol reagent (Invitrogen Life Technologies, Grand Island, NY, USA) and one μg of RNA was reverse transcribed into complementary DNA (cDNA) (Advantage RT-for-PCR Kit, Clontech, Palo Alto, CA, USA) according to the manufacturer’s instructions. Step One Real-Time PCR System (Applied Biosystems, Carlsbad, CA, USA) was used for amplification by real-time PCR, using FAM-labeled Taqman probes (Applied Biosystems, Thermo Fisher Scientific, Waltham, MA, USA), included Hs00164004_m1 (Collagen Ia1) and Hs00172187_m1 POLR2A (reference gene). All PCRs were carried out in a mixture of 25 μL, which contained four μL of cDNA (100 ng) and 12.5 μL of 2× PCR Master Mix (Applied Biosystems). The PCR conditions were 2 min at 94 °C followed by 40 cycles of 15 s at 95 °C and 60 °C 1 min concluding with an infinite loop of refrigeration. Results from two different experiments performed in triplicate are expressed as the mean ± SD of 2^^−ΔΔ*C*t^ of target gene normalized against POLR2A.

### 4.3. Detection of Apoptosis

Cell apoptosis was measured by using either the APOPercentage apoptosis assay kit (Biocolor Ltd., Northern Ireland, UK), or the Caspase-Glo 3/7 assay (Promega, Madison, IL, USA). The first one indirectly measures the phosphatidylserine transmembrane movement. Forty-eight well plates were seeded with 40,000 cells per well and incubated for the desired times in free serum medium. After the incubation period, three control wells were incubated with 5 mM H_2_0_2_ for 30 min (positive control). Cells were stained with the APOPercentage dye (100 µL) for 30 min at 37 °C in humidified air with 5% CO_2_. Cells were washed twice with PBS 500 µL and trypsinized (50 µL) for 10 min. Finally, 200 µL of dye release reagent were added to each well and the plate was shaken for 10 min and 100 µL aliquots of each well were transferred to a 96 well plate and absorbance was measured at 550 nm. Caspase-Glo 3/7 assay measures caspase-3 and -7 activities. Ninety-six well plates were seeded with 20,000 cells and incubated for the desired times in free serum medium. After the incubation period, conditioned medium was substituted with the Caspase-Glo reagent following the manufacturer recommendations. Luminescence was measured with the Filter Max Pro 5 Multi-Mode Microplate Reader (Molecular Devices, San Jose, CA, USA), results are reported as relative light units (RLU).

### 4.4. Cytosolic Ca^2+^ Measurements

The technique for intracellular Ca^2+^ signal measurement has been described elsewhere [8]. Briefly, cultured NHLF were loaded with 3 µM Fura-2/AM during 45 min at room temperature and then visualized by an upright epifluorescence microscope (Axiolab, Carl Zeiss, Oberkochen, Germany) equipped with a Zeiss 63× Achroplan objective. [Ca^2+^]_i_ was measured by calculating the ratio of the average fluorescence emitted at 510 nm when the cells were excited alternatively at 340 and 380 nm (ratio F340/F380). An elevation in [Ca^2+^]_i_ leads to an elevation in the Ratio F340/F380. Ratio measurements were carried out and plotted every 3 s by custom-made software. All recordings were carried out at room temperature (21–23 °C). In some experiments, U73122 (a widely employed PLC inhibitor) at 10 µM in dimethyl sulfoxide (DMSO) was used. Cells were pre-incubated for 15 min with this substance before adding the phospholipids and the corresponding controls had the same amount of DMSO as the experiments. The analysis of the Ca^2+^ data was performed by using ImageJ software (National Institutes of Health, Bethesda, MD, USA, http://rsbweb.nih.gov/ij/).

### 4.5. In Vivo Murine Model

C57/BL mice (8 weeks age, 20 g average weight) were used. All the experiments were performed according to protocols approved by the Animal Care and Use Committee of Animals of the Benemerita Universidad Autonoma de Puebla College of Medicine. Mice were anesthetized with Ketamine/Xylazine (100 mg·Kg^−1^/10 mg·Kg^−1^) and a single intratracheal instillation of saline (0.9%, controls) or saline containing bleomycin sulfate (4 U/Kg, Sigma-Aldrich) in a volume of 30 μL was administered.

All treatments were administered via aerosol as described elsewhere [37]. Briefly, mice were anesthetized and placed in 50 mL plastic tube with an orifice at the bottom where the nose was directed. The tube was connected to a micronebulizer (PARI GmbH, Starnberg, Germany) driven by an air line with a flow rate of 4 L/min. The final volume in the micronebulizer was adjusted to 1.5 mL in all cases. The tube was assembled in such a way that only the nose of the animal was in contact with the aerosol. All treatments were given every 48 h for a total of 6 doses starting on day one after bleomycin instillation.

### 4.6. Soluble Collagen Assay

Lungs were weighted, minced and agitated for 24 h in acetic acid 0.5 M 1:10 (*w*/*v*). Samples were filtered and soluble collagen content was measured using Sircol assay (Biocolor Ltd.), according to manufacturer specifications. Briefly, the contents of all tubes were adjusted to 100 µL with distilled water; a collagen standard was prepared with 100 mL aliquots containing 5, 10, 25 and 50 µg. 1 mL of Sircol Dye reagent was added and the content was mixed by inverting. Tubes were shaken mechanically for 30 min; during this time, collagen-dye complex precipitate out of solution. Samples were centrifuged 10,000× *g* for a 10 min period. The unbound dye solution was removed by carefully inverting and draining the tubes. One mL of Alkali reagent was added and vortexed. Samples were read at 540 nm in a spectrophotometer. Results are reported as µg of collagen/µg of lung tissue.

### 4.7. Histologic Examination

Lung sections from bleomycin-injured and control mice were stained, coded and scored blindly for percent of fibrosis as described elsewhere [38]. The presence of interstitial fibrosis was scored as follows: 0 = no fibrosis, 1 = up to 25% of the field, 2 = 25–50% of field, 3 = 50–75% of field and 4 = 75–100% of field. The assessment was done on the slide scanned completely in zigzag fashion, at 20× magnification. At least 20 fields were assessed in each slide. The mean and median of the scores for each animal was computed and the average of for each group was calculated for statistical comparison. The same pathologist evaluated the percentage of fibrosis twice on the same slides with a 6-month difference (intraclass correlation coefficient of 0.82, *p* < 0.05).

### 4.8. Preparation of Phospholipids

1,2-Dipalmitoyl-*sn*-glycero-3-phosphorylcholine (DPPC), l-α-Phosphatidyl-dl-glycerol (PG), 1,2-Dioleoyl-sn-glycero-3-phosphoethanolamine (PE) were purchased from Sigma-Aldrich (St. Louis, MO, USA). DPPC and PG were sonicated (CPX-400, Cole-Parmer, EUA, Vernon Hills, IL, USA) in Ham F12 medium at 4° C for 2 min. PE was re-suspended in chloroform at a concentration of 20 mg mL^−1^ and stored at −70° C. Prior to use, the desired amount of PE was evaporated with nitrogen, Ham F12 medium with 1% albumin was added to the pellet and sonicated as specified above.

### 4.9. Statistical Analysis

Unless otherwise stated, results are expressed as the mean ± SD of triplicates of two or three pooled experiments. For statistical analysis ANOVA or *t*-test was used; a *p* < 0.05 was considered significant. The software used was R version 3.3.3 with the multcomp package version 1.4-6 (R Foundation for Statistical Computing, Vienna, Austria). The data for all the experiments presented in this work, can be found at Supplementary Materials.

## Figures and Tables

**Table 1:** Treatment protocol of C57/BL mice.

Group	Intratracheal Bleomycin	Treatment	*n*
1	No (normal saline)	Normal saline (1 mL)	7
2	Yes (4 units Kg^−1^)	Normal saline (1 mL)	7
3	Yes (4 units Kg^−1^)	Beractant (400 mg·Kg^−1^)	7
4	Yes (4 units Kg^−1^)	Beractant-PE (400 mg·Kg^−1^) *	6
5	Yes (4 units Kg^−1^)	PE (400 mg·Kg^−1^)	5

* Beractant-PE: Beractant enriched with PE sonicated in normal saline at a final concentration of 0.75 mg/mL.

## Supplementary Materials

Supplementary materials can be found at http://www.mdpi.com/1422-0067/19/9/2758/s1.

## Abbreviations

0Ca^2+^	Removal of extracellular Ca^2+^
2-APB	2-Aminoethoxydiphenyl borate
CPA	Cyclopiazonic acid
DMSO	Dimethyl sulfoxide
DPPC	Dipalmitoilphosphatidilcoline
ER	Endoplasmic reticulum
InsP_3_	Inositol-1,4,5-trisphosphate
IPF	Idiopathic pulmonary fibrosis
NHLF	Normal human lung fibroblasts
PC	Phosphatidylcholine
PE	Phosphatidylethanolamine
PG	Phosphatidylglycerol
PL	Phospholipids
PLC	Phospholipase C
RLU	Relative Light Units
SERCA	Sarco-endoplasmic reticulum Ca^2+^-ATPase
SOCE	Store-operated Ca^2+^ entry
